# Unambiguous Acquisition/Tracking Technique Based on Sub-Correlation Functions for GNSS Sine-BOC Signals

**DOI:** 10.3390/s20020485

**Published:** 2020-01-15

**Authors:** Fang Hao, Baoguo Yu, Xingli Gan, Ruicai Jia, Heng Zhang, Lu Huang, Boyuan Wang

**Affiliations:** 1 College of Automation, Harbin Engineering University, No.145, Nantong Street, Harbin 150001, China; haofang@hrbeu.edu.cn; 2 State Key Laboratory of Satellite Navigation System and Equipment Technology, Shijiazhuang 050081, China; yubg@sina.cn (B.Y.); jiaruicai@126.com (R.J.); 13582161539@163.com (H.Z.); 18642720668@163.com (L.H.); boyuan@hrbeu.edu.cn (B.W.); 3 The 54th Research Institute of China Electronics Technology Group Corporation, Shijiazhuang 050081, China

**Keywords:** Global Navigation Satellite System, BOC, acquisition, tracking, PRN code, unambiguous

## Abstract

The autocorrelation function (ACF) of the Binary Offset Carrier modulation (BOC) signal for Global Navigation Satellite System (GNSS) has multiple peaks, ambiguity is easily generated during the synchronization of the baseband signal. Some methods have been proposed to remove the ambiguity, but the performance is not suitable for high-order BOC signals or does not maintain narrow correlation characteristics. This paper proposes a sub-function reconstruction synchronization algorithm to solve this problem, of which the key is to design a new local auxiliary code: the local Pseudo-Random Noise (PRN) code is divided into several new codes with different delays. The auxiliary code performs a coherent integration operation with the received signal. Then, a correlation function without any positive side peaks is obtained by multiplying the two correlation results to make the acquisition/tracking completely unambiguous. The paper gives a design scheme of navigation signal acquisition/tracking and deduces the theoretical analysis of detection performance. The phase discrimination function is provided. The performance of the method is analyzed from both theoretical and simulation aspects. Compared with the Binary phase shift keying-like (BPSK-LIKE) method, Subcarrier Phase Cancellation (SCPC) method and the Autocorrelation Side-Peak Cancellation Technique (ASPeCT) method, the proposed method has the best detection probability for the acquisition, which is 0.5 dB-Hz better than ASPeCT. For tracking, the proposed method performs best in terms of phase-detection curve, anti-multipath performance, and anti-noise performance. For high-order BOC signals, the SRSA technique successfully removes the false lock points, and there is only one multipath error envelope, and the code tracking error is almost the same as the ASPeCT method.

## 1. Introduction

Compared to traditional the Global Navigation Satellite System (GNSS), the addition of BOC modulation signals marks a breakthrough in the development of modern GNSS. The frequency spectrum splitting characteristic of the BOC signal helps different GNSS signals efficiently utilize limited band resources. The ACF of the BOC modulation signal also has a narrow correlation main peak, and a large main peak slope allows the signal to have better confidentiality and location accuracy. However, there is a severe ambiguity in the synchronization processing of BOC signal, because the BOC signal has not only the spectral splitting characteristics, but is also characterized by correlation function multi-peak. Only the main peak is the correct sync position of the corresponding signal, while other side peaks will cause false acquisition and mis-locking of the BOC signal. Although the BOC modulation signal possesses stronger anti-multipath performance compared with the conventional BPSK modulation signal, the multipath error is still the primary source of localization error of the BOC signal [1]. Therefore, how to remove the ambiguity of the BOC signal and enhance its anti-multipath performance has become a research hotspot in the field of navigation.

At present, there are three main types of processing methods for acquisition and tracking the BOC signal: The first type is to avoid the ambiguity detection method. These methods, such as the double estimation technique (DET) method [2,3] and the Bump-jump (BJ) method [4], detect the occurrence of error acquisition or mis-locking during the synchronization process of the BOC signal by adding a correlator. Because such methods require more extended detection and recovery times, they are only suitable for strong signal situations. The second category is the idea of degraded processing. This method uses the spectral relationship between BOC and BPSK to convert the BOC correlation function into a BPSK- LIKE correlation function with only a single peak, such as the BPSK-LIKE method [5]. The correlation main peak width obtained by this method is the same as for BPSK, which sacrifices the advantages of anti-multipath and high precision brought by the narrow correlation characteristics of the BOC signal. The third type is the idea of combining a single narrow main peak. These methods achieve the elimination or suppression of the sub-peak through a particular combination algorithm by locally generating a unique auxiliary code, and retains or generates a new single narrow main peak, such as ASPeCT [6], SCPC [7], GRASS [8] and PUDLL [9]. The ASPeCT method combines an unambiguous new function by the ACF of the BOC signal and its cross-correlation function (CCF) with the PRN code. But it only applies to the BOC(*n*, *n*) signal. The SCPC method performs coherent integration by receiving a signal with a local BOC signal modulated by two orthogonal subcarriers to obtain an unambiguous new function. Although this method is applicable to BOC(*m*, *n*) signals, it does not retain the narrow correlation characteristics of BOC signals. PUDLL [9] and GRASS [8] design some local auxiliary signals and generated a new correlation function with no side-peaks. However, as the PUDELL method increases with the order of BOC modulation, the code tracking performance decreases rapidly, and the GRASS method sacrifices performance. These methods take advantage of the BOC signal and become a vital research program.

This paper proposes a sub-function reconstruction synchronization algorithm (SRSA), which can solve the ambiguity. The structure of the acquisition and tracking algorithm is designed. The corresponding unambiguous non-coherent discrimination function for tracking process and the principle of combination are presented. The acquisition/tracking performance of this algorithm is analyzed from both theoretical and simulation aspects. Comparing BPSK-LIKE, SCPC, and ASPeCT with the proposed algorithm, simulation experiments show that the proposed algorithm can remove the interference of side peaks while retaining the narrow main peak characteristics of the correlation function. And code tracking accuracy and acquisition sensitivity have been significantly improved.

The organization of this paper is as follows. Section 2 presents the ambiguity problem, defines a new local auxiliary signal, and puts forward an algorithm for solving the ambiguity problem. In Section 3, an unambiguous acquisition scheme is provided, and the acquisition performance of the proposed algorithm is theoretical and simulated. Section 4 demonstrates tracking methods and the phase discrimination function. The tracking performance of the proposed algorithm is analyzed from three aspects of phase discrimination, anti-multipath, and anti-noise. And the final section draws a conclusion.

## 2. The Combination Principle of SRSA

### 2.1. The Characteristics of BOC(Kn,n) Correlation Function and Problems

According to the BOC modulation principle, BOC(*kn*, *n*) can be expressed as the following:

(1) $$S_{BOC\left(kn,n\right)}\left(t\right)=A\cdot D\left(t\right)\cdot c\left(t\right)\cdot sign\left(\sin\left(2\pi f_{sc}t\right)\right)$$

In the above formula, A represents the signal amplitude; D(t) represents the navigation signal data code; c(t) is PRN code; Subcarriers are represented by $sign\left(\sin\left(2\pi f_{sc}t\right)\right)$. Using $f_{0}=1.023\;\mathrm{MHz}$ as the reference frequency, BOC(*kn*, *n*) has the characteristics of PRN code rate $f_{c}=nf_{0}$ and subcarrier frequency $f_{sc}=knf_{0}$. N=2kn/n=2k is defined as the modulation factor of BOC(*kn*, *n*). The BOC(*kn*, *n*) code after PRN code and subcarrier modulation can be expressed as:

(2) $$S_{BOC}\left(t\right)=c\left(t\right)\cdot sign\left(\sin\left(2\pi f_{sc}t\right)\right)$$

**Figure 1:**
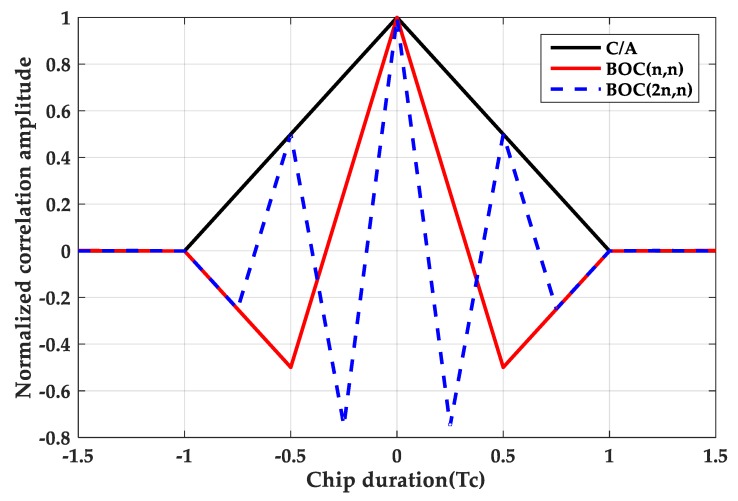
Auto-correlation function.

**Figure 2:**
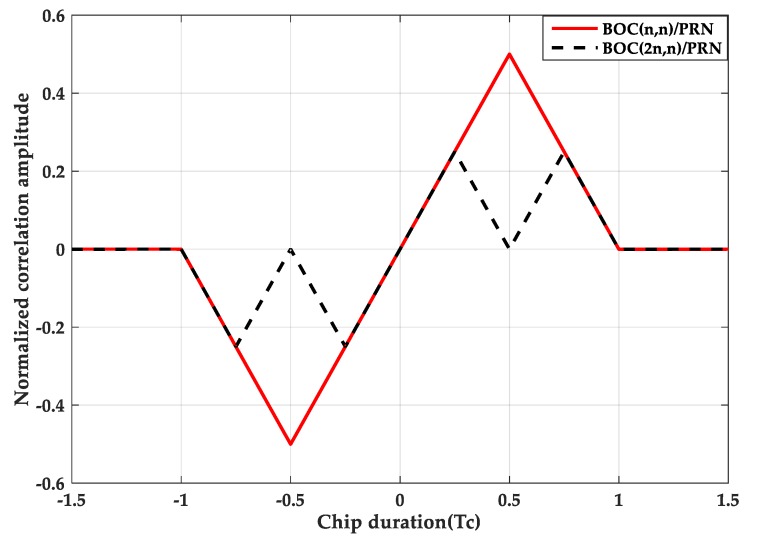
Cross-correlation functions.

Since the PRN code and subcarrier have specific correlation characteristics, the correlation function of the BOC(*kn*, *n*) signal has multiple peaks. The mathematical formula of the autocorrelation function $R_{B}\left(\tau \right)$ and the cross-correlation function $R_{B/P}\left(\tau \right)$ of the BOC(*kn*, *n*) is [10]

(3) $$R_{B}\left(\tau \right)=\sum_{i=1}^{N-1}\left\{\left[{\left(-1\right)}^{i}tri\left(\tau +\frac{T_{c}}{N}\right)+{\left(-1\right)}^{i}tri\left(\tau -\frac{T_{c}}{N}\right)\right]\times \frac{N-i}{N}\right\}+tri_{0}\left(\tau \right)$$

(4) $$R_{B/P}\left(\tau \right)=\sum_{i=1}^{N/2}\left\{\left[tri\left(\tau +\frac{\left(2i-1\right)T_{c}}{N}\right)+\left(-1\right)tri\left(\tau -\frac{\left(2i-1\right)T_{c}}{N}\right)\right]\times \frac{1}{N}\right\}$$

where $tri\left(\tau -aT_{c}\right)$ represents a triangular function with peak at a·Tc. The triangle duration is ${2T}_{sc}$, and $T_{sc}=T_{c}/N$. $Tc=1/f_{c}$ is a PRN code chip duration. τ indicates the code phase delay. Figure 1 is a comparison of autocorrelation functions of BPSK, BOC(*n*, *n*) and BOC(2*n*, *n*). Figure 2 is a comparison of the cross-correlation functions between the BOC(*n*, *n*)/BOC(2*n*, *n*) signals and the PRN code. As can be seen from the figures, the larger the modulation factor, the greater the number of correlation peaks.

**Figure 3:**
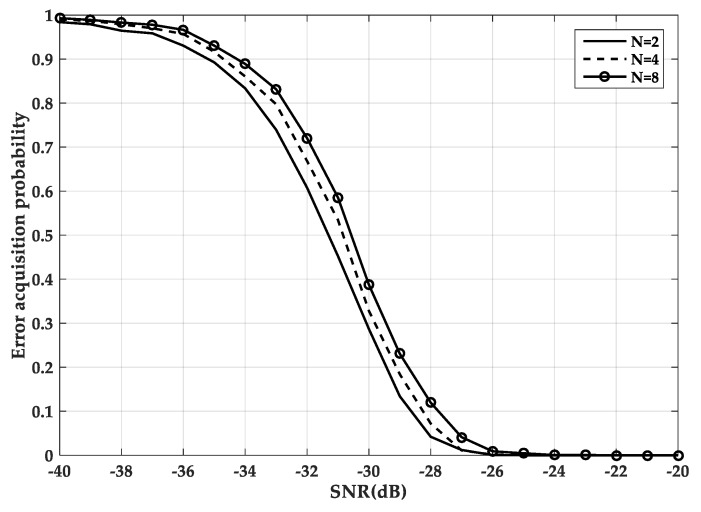
The false acquisition probability of three different factors.

However, for BOC signals, once the side peaks are acquired, the false locks will occur during the subsequent tracking phase [11]. Especially for high-order BOC signals, it is difficult to distinguish the main peak and the sub-peak of the correlation function, which is more likely to cause the false lock phenomenon. The relationship between the false acquisition probability of different factors N and the signal-to-noise ratio (SNR) is given in Figure 3. SNR is defined as the ratio of signal to noise in an electronic device or electronic system. It can be seen that in the same SNR environment, the larger the modulation factor, the greater the probability of false acquisition. At −27 dB, the false acquisition probability of N = 4 and N = 2 are the same, close to zero, and the false acquisition probability of N = 8 is still 0.05.

In the code tracking stage, the classic EMLP (Early-Minus-Late-Power) phase discriminator is used, and the phase discrimination formula is

(5) $$V\left(\Delta \tau \right)=R_{B}^{2}\left(\Delta \tau -d/2\right)-R_{B}^{2}\left(\Delta \tau +d/2\right)$$

**Figure 4:**
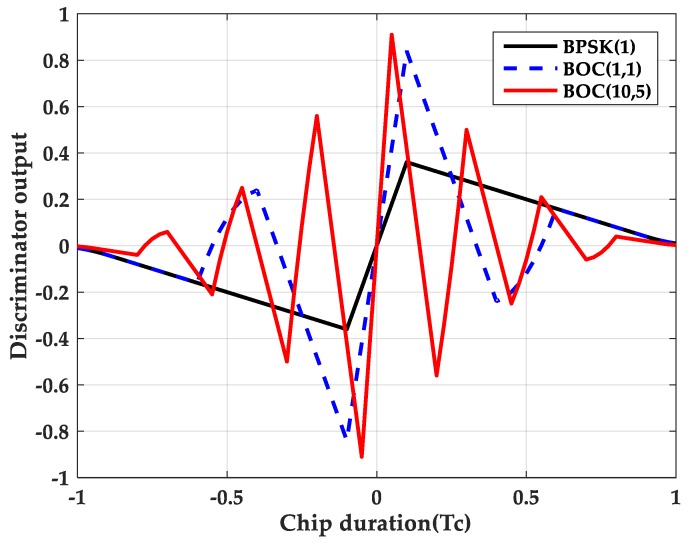
The discrimination curves for the three methods.

Δτ represents the estimation error of the code phase. *d* is the delay interval between the early and late correlators. Figure 4 shows the discrimination curves for the BPSK signal, the BOC(1,1) signal, and the BOC(10, 5) signal in an infinite bandwidth. False-lock point corresponds to a zero of the discriminator where the S-curve presents a positive slope. Obviously, the larger the modulation factor, the more false-lock points, which are generated by the side peaks of ACF. This situation will result in intolerable code tracking errors.

### 2.2. Generation of Sub-PRN Code Sequences

By definition, the BOC code and the PRN code are expressed in the form of pulses as follows.

(6) $$S_{PRN}(t)=\sum_{i=-\infty }^{\infty }C_{i}P_{T_{C}}\left(t-iT_{C}\right)=\sum_{i=0}^{M}S_{PRN,i}\left(t\right)$$

(7) $$s_{BOC}(t)=\sum_{j=0}^{M-1}\sum_{i=-\infty }^{\infty }C_{i}{\left(-1\right)}^{j}P_{T_{sc}}(t-iT_{C}-jT_{sc})$$

**Figure 5:**
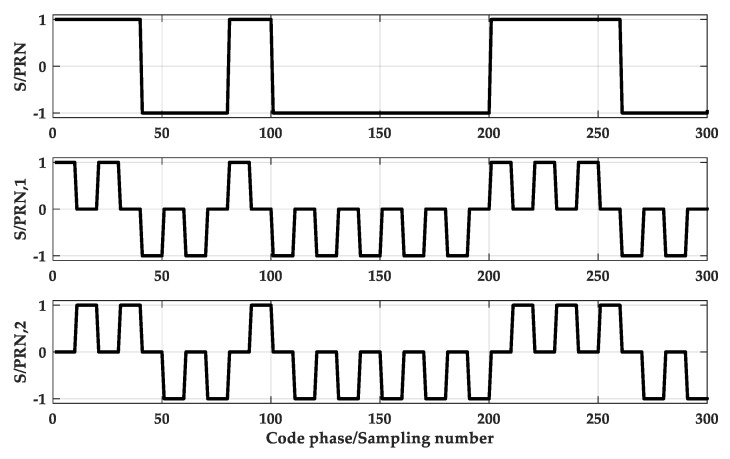
The Separation of PRN at M = 2.

**Figure 6:**
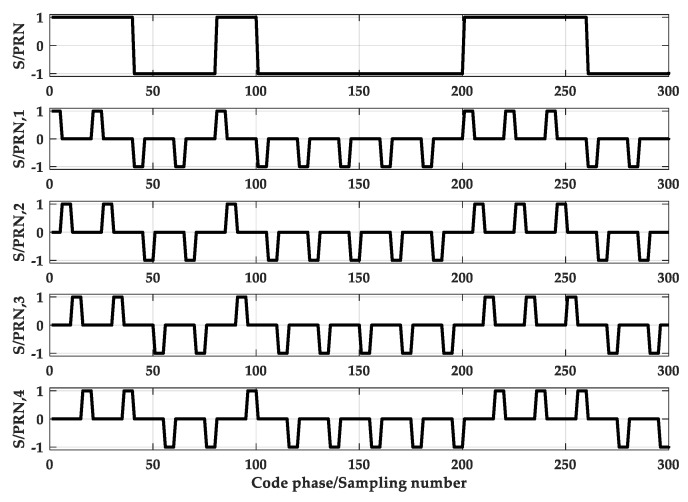
The Separation of PRN at M = 4.

$P_{T_{C}}$ is a rectangular pulse with a period of $T_{C}$ and an amplitude of 1. $P_{T_{sc}}$ is a rectangular pulse with a duration of $T_{sc}$ and an amplitude of 1, and $T_{sc}=T_{c}/N$.$C_{i}$ is the symbol of the chip, $C_{i}\in \left(-1,1\right)$. M is the total number of pulses in a chip interval, which means that each PRN code chip is divided into M segments. Here M = N. This paper uses $S_{PRN,i}\left(t\right)$ to represent the *i*-th sub-PRN code. Then, separate the local PRN code, retain the *i*-th segment, and set the rest to zero. When M = 2, as shown in Figure 5, when M = 4, as shown in Figure 6.

It is supposed that the coherent integration time is $T_{coh}$, then the cross-correlation function of BOC code and PRN code is:

(8) $$\begin{array}{cc} R_{B/P}\left(\tau \right) & =\frac{1}{T_{coh}}\int_{0}^{T_{coh}}S_{BOC}\left(t\right)\cdot S_{PRN}\left(t+\tau \right)dt\\ & =\frac{1}{T_{coh}}\frac{T_{coh}}{T_{c}}\int_{-\tau }^{T_{c}}S_{BOC}\left(t\right)\cdot S_{PRN}\left(t+\tau \right)dt\\ & =\sum_{i=1}^{M}R_{B/Pi}\left(\tau \right) \end{array}$$

(9) $$R_{B/Pi}\left(\tau \right)=\frac{1}{T_{coh}}\int_{0}^{T_{coh}}S_{BOC}\left(t\right)\cdot S_{PRN,i}\left(t+\tau \right)dt$$

**Figure 7:**
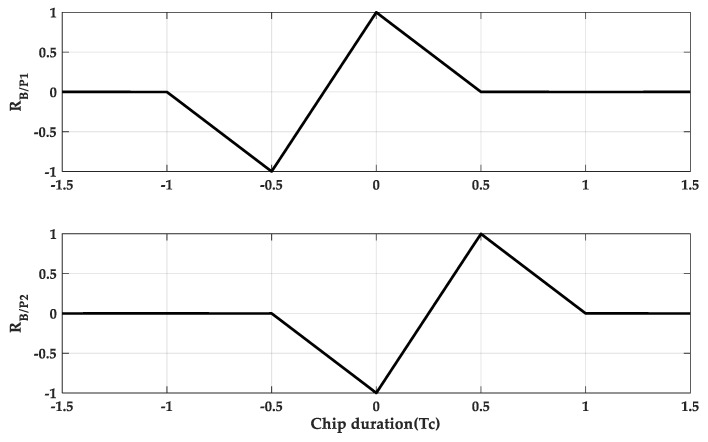
The normalized sub cross-correlation functions at M = 2.

**Figure 8:**
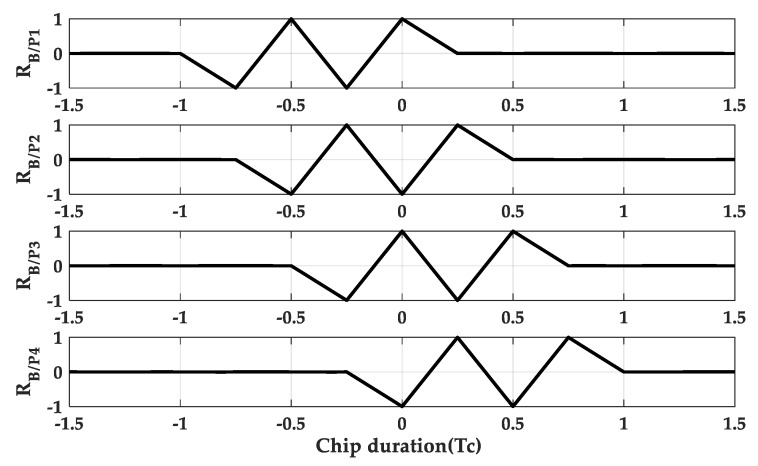
The normalized sub cross-correlation functions at M = 4.

Figure 7 is a schematic diagram of sub-code separation for M = 2. And Figure 8 is a schematic diagram of sub-code separation for M = 4.

And it can also be represented by triangular functions. When M = 2, it can be expressed as

(10) $$R_{B\left(n,n\right)/p1}=\frac{1}{2}\sum_{i=1}^{1}tri\left(\tau +\left(2i-1\right)Tsc-Tsc\right)-tri\left(\tau -\left(2i-1\right)Tsc\right)$$

(11) $$R_{B\left(n,n\right)/p2}=\frac{1}{2}\sum_{i=1}^{1}tri\left(\tau +\left(2i-1\right)Tsc\right)-tri\left(\tau -\left(2i-1\right)Tsc+Tsc\right)$$

And when M = 4 and the triangle duration is ${2T}_{sc}$, it can be expressed as

(12) $$R_{B\left(2n,n\right)/p1}=\frac{1}{4}\sum_{i=1}^{2}tri\left(\tau +\left(2i-1\right)Tsc-3Tsc\right)-tri\left(\tau -\left(2i-1\right)Tsc\right)$$

(13) $$R_{B\left(2n,n\right)/p2}=\frac{1}{4}\sum_{i=1}^{2}tri\left(\tau +\left(2i-1\right)Tsc-2Tsc\right)-tri\left(\tau -\left(2i-1\right)Tsc+Tsc\right)$$

(14) $$R_{B\left(2n,n\right)/p3}=\frac{1}{4}\sum_{i=1}^{2}tri\left(\tau +\left(2i-1\right)Tsc-Tsc\right)-tri\left(\tau -\left(2i-1\right)Tsc+2Tsc\right)$$

(15) $$R_{B\left(2n,n\right)/p4}=\frac{1}{4}\sum_{i=1}^{2}tri\left(\tau +\left(2i-1\right)Tsc\right)-tri\left(\tau -\left(2i-1\right)Tsc+3Tsc\right)$$

### 2.3. Correlation Function Combination

By analyzing the sub-correlation functions of the BOC(*kn*, *n*) signal with each modulation factor, it is found that the main peak position of the correlation function of the first segment corresponds to the main peak position of the correlation function of the *M*-th segment, and the side peak corresponds to the zero points. No matter what value M is, the product of $R_{B/P1}$ and $R_{B/PM}$ can yield non-zero value only near the (0,0) point. And $R_{B/PM}$ can be obtained by $R_{B/P1}$ shift (M−1)Tc/M and multiply by −1. Thus, combining correlation functions, such as formulas

(16) $$R_{0}=-R_{B/P1}\cdot R_{B/PM}$$

It can be found that $R_{0}$ has two small sub-peaks with a negative value and a positive main peak. To achieve the purpose of eliminating sub-peaks, the combination algorithm is

(17) $$R_{SRSA}=\left|\left|R_{B/P1}\cdot R_{B/PM}\right|+R_{B/P1}\cdot R_{B/PM}\right|$$

**Figure 9:**
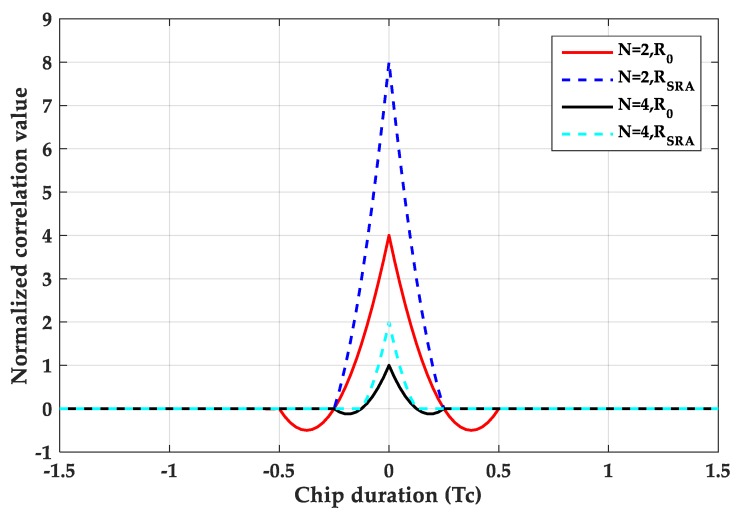
The combination results of correlation functions by SRSA.

Figure 9 is a simulation diagram of the combination function for M = 2 and M = 4. It can be seen from the graph that the larger M is, the smaller the main peak width of the combination function is.

## 3. Acquisition Structure Design and Performance Analysis Based on SRSA Principle

### 3.1. The Acquisition Structure Diagram

**Figure 10:**
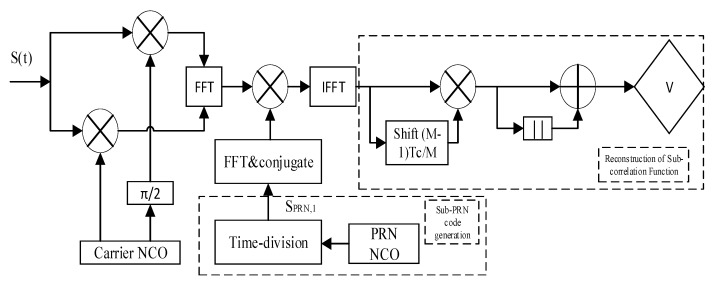
The SRSA acquisition schematic.

When the receiver generates a local signal, the local PRN sequence is separated to obtain a sub-signal. The sub-signal is correlated with the received signal to obtain a sub-correlation function, and the sub-correlation function is combined according to Equation (17) to get a correlation function of the no secondary peak. Figure 10 shows the SRSA acquisition schematic. It is worth noting that the sub-correlation functions can be obtained from each other. According to this property, only one sub-correlation function can be generated, and the generated sub-correlation function can be delayed to get other functions.

The steps for signal acquisition are as follows:

Step 1: The carrier NCO generates two orthogonal local carriers, and mixes with the received intermediate frequency (IF) BOC(*kn*, *n*) signal with the purpose of cancelling the carrier.

Step 2: Generate a local PRN code. And then generate a local auxiliary code according to the method in Section 2.2.

Step 3: The auxiliary code is coherently integrated with the input signal to obtain a sub-correlation function $R_{B/P1}$.

Step 4: Delay $R_{B/P1}$ by sample points of (M−1)/M PRN chip length, and a new sub-function $R_{B/PM}$ is obtained.

Step 5: According to the Formula (15), the final algorithm operation is performed to obtain a correlation function without a sub-peak, that is, the detection value V.

### 3.2. Detection Performance Analysis

The input BOC signal is expressed as follows:

(18) $$S(t)=\sqrt{2}A\times C\left(t-\tau \right)\times D\left(t-\tau \right)\times S_{C}\left(t-\tau \right)\times \cos\left(2\pi \left(f_{IF}+\Delta f_{D}\right)t+\varphi \right)+N_{n}$$

$\sqrt{2}A$ is the amplitude of the input signal, C(t) is the PRN code, D(t) is the navigation data, τ is the code delay of the input signal, ${\Delta f}_{D}$ is the Doppler frequency of the input signal, $f_{IF}$ is the intermediate frequency, $S_{c}(t)$ is the subcarrier, φ is the initial phase of carrier and $N_{n}$ is the noise term.

Mixing input signal with local carrier. For simplicity, we exploit the baseband complex envelope expression of $S_{O}(t)$.

(19) $$S_{O}(t)=\sqrt{2}A\times C\left(t-\tau \right)\times D\left(t-\tau \right)\times S_{C}\left(t-\tau \right)\times e^{j\left(2\pi \Delta f_{D}t+\varphi \right)}+N_{c}+jN_{s}$$

$N_{c}$ and $N_{s}$ are independent Gaussian random processes. They have the same zero mean and double-sided power spectrum density $N_{0}$. Then, multiply the local auxiliary signal according to the SRSA principle.

(20) $$S_{1}(t)\;=\sqrt{2}A\times C\left(t-\tau \right)\times D\left(t-\tau \right)\times S_{C}\left(t-\tau \right)\times S_{PRN,1}\left(t\right)\times e^{j\left(2\pi \Delta f_{D}t+\varphi \right)}+N_{c}+jN_{s}$$

(21) $$S_{M}(t)\;=\sqrt{2}A\times C\left(t-\tau \right)\times D\left(t-\tau \right)\times S_{C}\left(t-\tau \right)\times S_{PRN,M}\left(t\right)\times e^{j\left(2\pi \Delta f_{D}t+\varphi \right)}+N_{c}+jN_{s}$$

Then perform coherent integration, D(t) is a constant, does not affect the overall process, omitted here, no need to consider the change of navigation data bits.

(22) $$\begin{array}{l} {\bar{S}}_{1}=A\times R_{B/P,1}\left(\tau \right)\times \sin c\left(\pi \Delta f_{D}T_{coh}\right)\times e^{j\left(2\pi \Delta f_{D}T_{coh}+\varphi \right)}+N_{1}\\ \;\;\;\;\;\;\;\;\;\;\approx A\times R_{B/P,1}\left(\tau \right)\times \sin c\left(\pi \Delta f_{D}T_{coh}\right)+N_{1}\\ \;\;\;\;\;\;\;\;\;\;={\bar{S}}_{1}\left(\tau ,\Delta f_{D}\right)+N_{1} \end{array}$$

(23) $$\begin{array}{l} {\bar{S}}_{M}=A\times R_{B/P,M}\left(\tau \right)\times \sin c\left(\pi \Delta f_{D}T_{coh}\right)\times e^{j\left(2\pi \Delta f_{D}T_{coh}+\varphi \right)}+N_{m}\\ \;\;\;\;\;\;\;\;\;\;\;\;\;\approx A\times R_{B/P,M}\left(\tau \right)\times \sin c\left(\pi \Delta f_{D}T_{coh}\right)+N_{m}\\ \;\;\;\;\;\;\;\;\;\;\;\;\;={\bar{S}}_{M}\left(\tau ,\Delta f_{D}\right)+N_{m} \end{array}$$

When the carrier and code loop tracking is successful, the complex exponential is almost 1. ${\bar{S}}_{1}\left(\tau ,\Delta f_{D}\right)$ is the signal part of ${\bar{S}}_{1}$, ${\bar{S}}_{M}\left(\tau ,\Delta f_{D}\right)$ is the signal part of ${\bar{S}}_{M}$, and the non-coherent accumulation time is $M_{noh}$. In digital signal processing and communication theory, the normalized sinc function is usually defined as sinc(x)=sin(πx)/πx. According to the proposed combination principle, the final detection value is

(24) $$\begin{array}{ccc} S_{final} & = & \sum_{i=1}^{M_{noh}}\left(\left|\bar{S_{1}}\times \bar{S_{M}}\right|+\bar{S_{1}}\times \bar{S_{M}}\right)\\ & \approx & 2\sum_{i=1}^{M_{noh}}\left(\left|\bar{S_{1}}\times \bar{S_{M}}\right|\right)\\ & = & 2\sum_{i=1}^{M_{noh}}\left(\left|\bar{S_{1}}\left(\tau ,\Delta f_{D}\right)+N_{1}\right|\times \left|\bar{S_{M}}\left(\tau ,\Delta f_{D}\right)+N_{m}\right|\right)\\ & = & 2\sum_{i=1}^{M_{noh}}\left(\left|\bar{S_{1}}\left(\tau ,\Delta f_{D}\right)\right|\times \left|\bar{S_{M}}\left(\tau ,\Delta f_{D}\right)\right|\right)\\ & & +2\sum_{i=1}^{M_{noh}}\left(\left|\bar{S_{1}}\left(\tau ,\Delta f_{D}\right)\right|\times N_{m}\right)\\ & & +2\sum_{i=1}^{M_{noh}}\left(\left|\bar{S_{M}}\left(\tau ,\Delta f_{D}\right)\right|\times N_{1}\right)\\ & & +\;2\sum_{i=1}^{M_{noh}}\left(N_{1}\times N_{m}\right) \end{array}$$

The main peak of the correlation function is much higher than the side peak. $\left|\bar{S_{1}}\times \bar{S_{M}}\right|+\bar{S_{1}}\times \bar{S_{M}}$ can be approximated as $2\left|\bar{S_{1}}\times \bar{S_{M}}\right|$. Assuming that $X_{0}$ is the case that only noise presents in the received signal, $X_{1}$ is the other case that both signal and noise exist, here the two cases are analysed [12]. When the target signal does not exist, it can also be said that the received signal is phase-aligned with the local signal code outside of one chip. At this time, the detection statistic value $V_{1}$ obeys Rayleigh distributions. Suppose the probability density function (PDF) at this time is $P_{V_{1}}$, and the false alarm probability can be expressed as

(25) $$P_{fa}=P[V_{1}>V]=\int_{V}^{+\infty }P_{V_{1}}\left(x\right)dx=\int_{V}^{+\infty }\frac{V_{1}}{{\left(\sigma_{n}\right)}^{2}}\cdot \exp\left\{-\frac{V_{1}{}^{2}}{2{\left(\sigma_{n}\right)}^{2}}\right\}dV_{1}$$

Therefore, according to Equation (25), the threshold value V can be reversed after the false alarm probability and noise power are given. $\sigma_{n}{}^{2}=C/\left(10^{0.1\mathrm{dB}}\right)/0.004$. C is carrier power of the input (IF) signal. dB is the carrier-to-noise ratio range.

When the received signal is aligned with the locally generated chip, the detected value obeys the Rice distribution, and its mean and variance are

(26) $$E\left(V_{2}\right)=4M_{noh}A^{2}T_{coh}{}^{2}\left|R_{B/P1}\left(\tau \right)\right|\left|R_{B/PM}\left(\tau \right)\right|$$

(27) $$D\left(V_{2}\right)=16M_{noh}\sigma^{4}+\sum_{l=0}^{M_{noh}}\left[4A^{2}T_{coh}{}^{2}\left({\left|R_{B/P1}\left(\tau \right)\right|}_{l}\times {\left|R_{B/PM}\left(\tau \right)\right|}_{l}\right)\right]$$

when $M_{noh}$ is large, the detection statistic $V_{2}$ is approximately obeying the Rice distribution.

$P_{V_{2}}(x)$ is assumed to be the probability density at this time, and the detection probability $P_{d}$ is:

(28) $$P_{d}=\int_{V}^{+\infty }P_{V_{2}}\left(x\right)dx$$

(29) $$P_{V_{2}}\left(x\right)=\frac{V_{2}}{\sigma^{2}}\exp\left\{-\frac{V_{2}{}^{2}+a^{2}}{2\sigma^{2}}\right\}I_{0}\left(\frac{aV_{2}}{\sigma^{2}}\right)$$

$a^{2}/\sigma^{2}$ is the signal-to-noise ratio (SNR), $I_{0}(x)$ is the Modified Bessel function of the first kind. The detection statistics can only be maximized when the code phase error τ is close to zero.

### 3.3. Performance Simulation and Analysis

Based on the Matlab platform, the intermediate frequency of the input signal is 4.092 MHz, the sampling rate is set to 40.92 MHz, the code phase offset is in the 601th sampling point, the Doppler is set to 2000 Hz. The coherent integration time is $T_{coh}=1\;\mathrm{ms}$. The four acquisition methods of SRSA, SCPC, ASPeCT and BPSK-LIKE are compared to the performance.

#### 3.3.1. Disambiguation Performance Analysis

**Figure 11:**
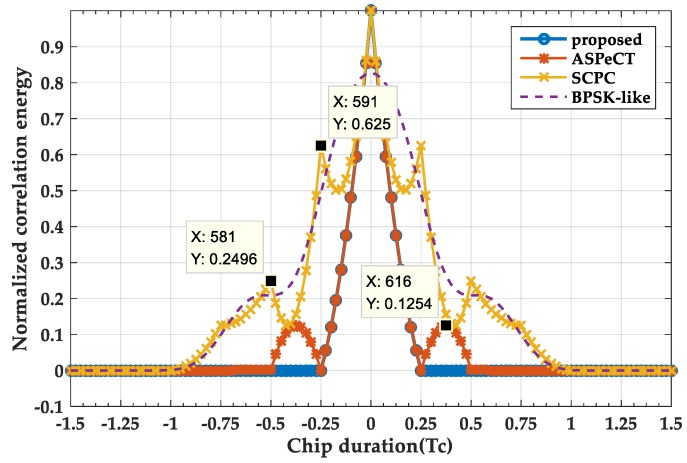
The 2-D acquisition result at M = 2.

**Figure 12:**
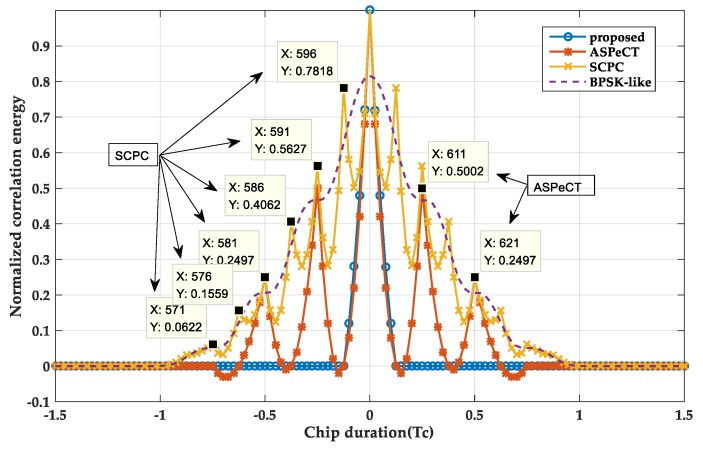
The 2-D acquisition result at M = 4.

Figure 11 and Figure 12 shows a normalized two-dimensional acquisition comparison of the four methods. When M = 2, it can be found that the SRSA method is better than the SCPC, ASPeCT, and BPSK-LIKE methods. In terms of main peak span, the SRSA main peak spans 20 sampling points (half chip points) and is the same as the ASPeCT method, and is better than the BPSK-LIKE method and the SCPC method 80 sampling points (i.e., two-chip points). For the peak value, the SRSA method is the same as the ASPeCT and SCPC methods and 10% higher than the BPSK-LIKE method. And the SRSA method removes the effects of side peaks while the ASPeCT still has two smaller sub-peaks. When M = 4, the SCPC and BPSK-LIKE methods remove the sub-peaks, but still do not retain the narrow correlation characteristic of BOC signal. The ASPeCT method has more sub-peaks of the correlation function when M = 4, which is not applicable to BOC signals above the 2nd order. And the SRSA can still completely remove all the sub-peaks of the 4th-order BOC, and the main peak span is only ten sampling points.

#### 3.3.2. Computational Complexity Analysis

The one millisecond signal is received in the pre-set simulation environment, and the number of frequency searches is 41 times. Using the FFT parallel code phase search method, the number of complex additions required to complete a 40,920 points FFT is:

(30) $$n\log_{2}^{n}=40,920\log_{2}^{40,920}=626,916$$

The number of complex multiplications required is

(31) $$\frac{n}{2}\log_{2}^{n}=\frac{40,920}{2}\log_{2}^{40,920}=313,458$$

One complex multiplication is equal to four real multiplications and two real additions, and a complex addition equals two real additions [13,14]. Table 1 is the computational complexity of the four algorithms in the acquisition. It shows from the table that the computational complexity of SRSA is the same as ASPeCT, and is better than BPSK-Like and SCPC.

#### 3.3.3. Acquisition Probability Analysis

**Figure 13:**
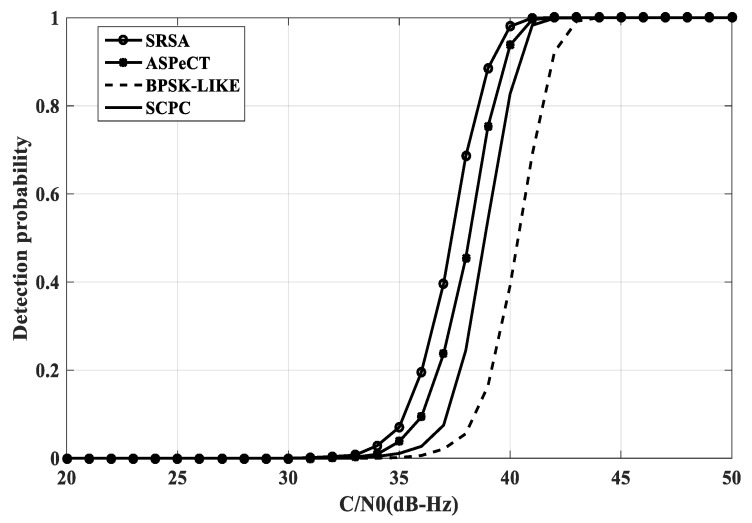
The acquisition probability of BOC(1, 1).

**Figure 14:**
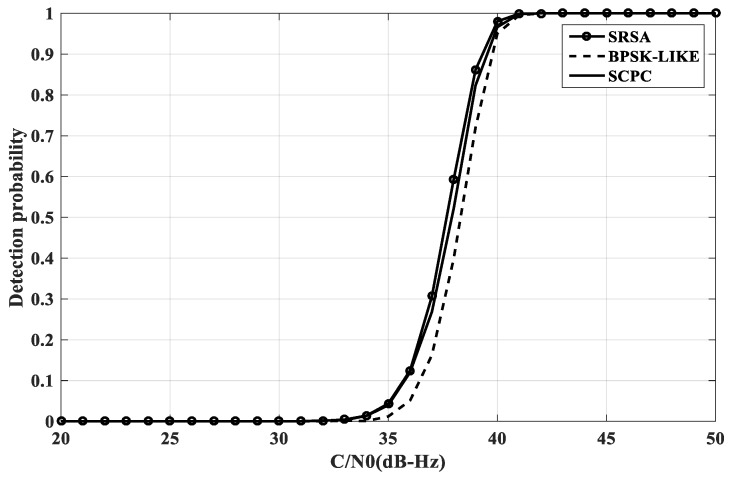
The acquisition probability of BOC(10, 5).

The Monte Carlo method is used to simulate the detection probability of BOC(1, 1) and BOC(10, 5) under different acquisition methods. Set the carrier-to-noise ratio range of the simulation environment to [20-50], set the false alarm probability, the non-coherent accumulation number is L = 10, and perform 2000 statistics as shown in Figure 13 and Figure 14.

As can be seen from the above figure, the SRSA method has obvious advantages when the acquisition probability is higher than 0.9. For 2nd order BOC signals, the SRSA acquisition probability is better than the BPSK-LIKE, SCPC and ASPeCT methods 2.5 dB-Hz, 1 dB-Hz, and 0.5 dB-Hz. For high-order BOC signals, the ASPeCT method cannot suppress the sub-peaks of the correlation function, and the effect is not satisfactory. There is no comparison here. The SRSA acquisition probability is better than the BPSK-LIKE and SCPC methods 0.5 dB-Hz and 0.25 dB-Hz when the acquisition probability is higher than 0.9.

#### 3.3.4. Peak-to-Average Ratio Analysis

**Figure 15:**
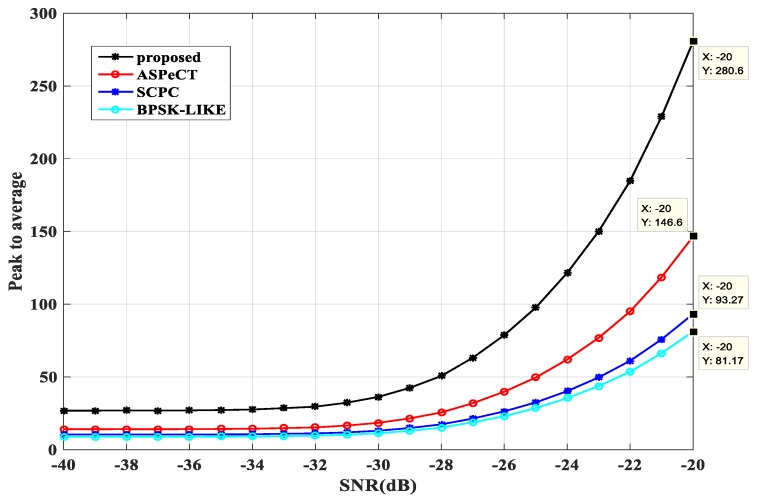
The peak to average ratio at M = 2.

**Figure 16:**
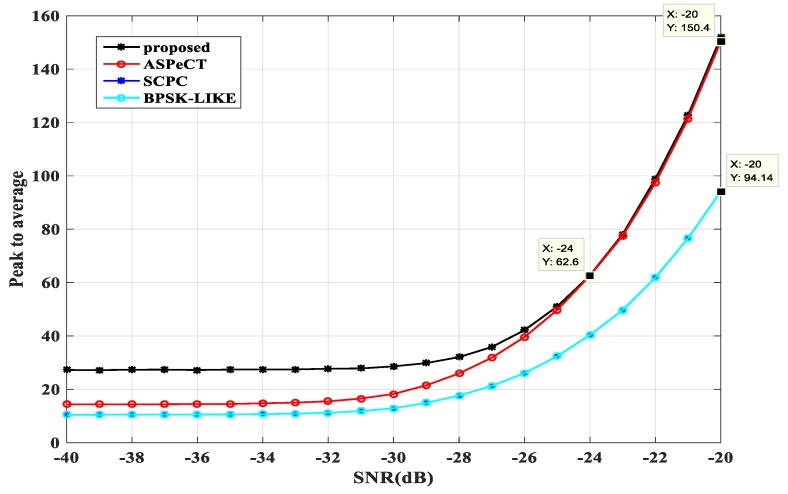
The peak to average ratio at M = 4.

The peak-to-average ratio is also an important indicator for evaluating the performance of an algorithm, and is defined as the ratio of the peak value to the average value of the correlation result obtained by the acquisition. In the preset simulation environment, the BPSK-LIKE, SCPC, ASPeCT and SRSA methods were compared by repeating 3000 experiments, and the peak-to-average ratios of the four methods at different signal-to-noise ratios were obtained, as shown in Figure 15 and Figure 16.

When M = 2, it can be seen that the performance of the SRSA method is optimal. At −20 dB, the value of the SRSA method is 3.5 times that of the BPSK-LIKE method, three times that of the SCPC method, and twice that of the ASPeCT method. The values of the four methods SRSA, ASPeCT, SCPC, and BPSK-LIKE are 280.6, 146.6, 93.27 and 81.17 at −20 dB.

When M = 4, the curve of the SRSA method intersects the curve of the ASPeCT method at −24 dB. The curve of the SCPC method and the BPSK-LIKE method almost coincide. At −20 dB, the values of the four methods SRSA, ASPeCT, SCPC, and BPSK-LIKE are 150.6, 150.4, 90.14 and 90.14.

## 4. Tracking Loop Structure Design and Performance Analysis Based on SRSA Principle

### 4.1. Discriminator Function and Tracking Loop Structure

Using the first and the M-th sub-PRN codes as two local auxiliary codes in the tracking loop. Without considering multipath and interference, the BOC IF signal received by a GNSS receiver from a satellite can be expressed as [15]:

(32) $$r\left(t\right)=\sqrt{2P}\cdot D\left(t\right)\cdot S_{BOC}\left(t\right)\cdot \cos\left(2\pi f_{IF}t+\varphi_{0}\right)+n\left(t\right)$$

Among them, P is the total power of the received signal, D(t) is the navigation data code, $S_{BOC}\left(t\right)$ is defined in Equation (6), $f_{IF}$ is the intermediate frequency after down-converting the received signal, $\varphi_{0}$ is the initial phase of the carrier. n(t) is Band-limited white noise [16], and it has the zero mean and double-sided power spectrum density $N_{0}$.

After the carrier is cancelled, the I and Q signals perform coherent integration operations with the Early and Late local auxiliary codes, and the following is obtained:

(33) $$\{\begin{array}{c} I_{E1}+jQ_{E1}=\sqrt{2P}R_{B/P1}\left(\Delta \tau -\frac{d}{2}\right)e^{j\Delta \varphi }+n_{IE1}+jn_{QE1}\\ I_{EM}+jQ_{EM}=\sqrt{2P}R_{B/PM}\left(\Delta \tau -\frac{d}{2}\right)e^{j\Delta \varphi }+n_{IEM}+jn_{QEM}\\ I_{L1}+jQ_{L1}=\sqrt{2P}R_{B/P1}\left(\Delta \tau -\frac{d}{2}\right)e^{j\Delta \varphi }+n_{IL1}+jn_{QL1}\\ I_{LM}+jQ_{LM}=\sqrt{2P}R_{B/PM}\left(\Delta \tau -\frac{d}{2}\right)e^{j\Delta \varphi }+n_{ILM}+jn_{QLM} \end{array}$$

In the formula, the subscripts 1 and M indicate the local code number, and the subscripts E and L respectively indicate the Early and Late branches, and other branches are similarly represented. Δτ and Δφ respectively represent the estimation error of the code phase and the initial phase of the carrier, d is the delay spacing between the early and late correlators. All n are noise terms obeying the Gaussian distribution [16,17].

Combined with the Formulas (26) and (27), The distribution function of the outputs $I_{E1}$, $I_{EM}$, $I_{L1}$, $I_{LM}$ at Δτ=0 is

(34) $$\begin{array}{l} {\left(I_{E1},I_{EM},I_{L1},I_{LM}\right)}^{T}\sim N\left(\mu \cos\left(\Delta \varphi \right),\omega \right)\\ {\left(Q_{E1},Q_{EM},Q_{L1},Q_{LM}\right)}^{T}\sim N\left(\mu \sin\left(\Delta \varphi \right),\omega \right) \end{array}$$

with

(35) $$\mu =\sqrt{2P}\cdot \sin c\left(\pi \Delta fT_{coh}\right)\cdot {\left[R_{B/P1}\left(-d/2\right)\;\;\;\;\;R_{B/P1}\left(d/2\right)\;\;\;\;\;R_{B/PM}\left(-d/2\right)\;\;\;\;\;R_{B/PM}\left(d/2\right)\right]}^{T}$$

(36) $$\omega =\frac{N_{0}}{T_{coh}}\left[\begin{array}{cccc} R_{B}\left(0\right) & R_{B}\left(d\right) & R_{B/P1}\left(0\right) & R_{B/P1}\left(d\right)\\ R_{B}\left(d\right) & R_{B}\left(0\right) & R_{B/P1}\left(-d\right) & R_{B/P1}\left(0\right)\\ R_{B/PM}\left(0\right) & R_{B/PM}\left(-d\right) & R_{P1/PM}\left(0\right) & R_{P1/PM}\left(d\right)\\ R_{B/PM}\left(d\right) & R_{B/PM}\left(0\right) & R_{P1/PM}\left(d\right) & R_{P1/PM}\left(0\right) \end{array}\right]$$

The output of Q branch and I branch are independent of each other. The distribution function of the outputs $Q_{E1}$, $Q_{EM}$, $Q_{L1}$, $Q_{LM}$ at Δτ=0 is N(0,ω). $R_{P1/PM}$ is the cross-correlation function of the auxiliary signal.

According to Equations (17), the formula for the incoherent discriminator function is:

(37) $$\begin{array}{cc} D_{SRSA}\left(\Delta \tau \right) & =\left(\begin{array}{l} \left|\left(I_{E1}+Q_{E1}\right)\cdot \left(I_{EM}+Q_{EM}\right)\right|+\\ \left(I_{E1}+Q_{E1}\right)\cdot \left(I_{EM}+Q_{EM}\right) \end{array}\right)\\ & -\left(\begin{array}{l} \left|\left(I_{L1}+Q_{L1}\right)\cdot \left(I_{LM}+Q_{LM}\right)\right|+\\ \left(I_{L1}+Q_{L1}\right)\cdot \left(I_{LM}+Q_{LM}\right) \end{array}\right)\\ & =2P\left(R_{SRSA}^{2}\left(\Delta \tau -d/2\right)-R_{SRSA}^{2}\left(\Delta \tau +d/2\right)\right) \end{array}$$

**Figure 17:**
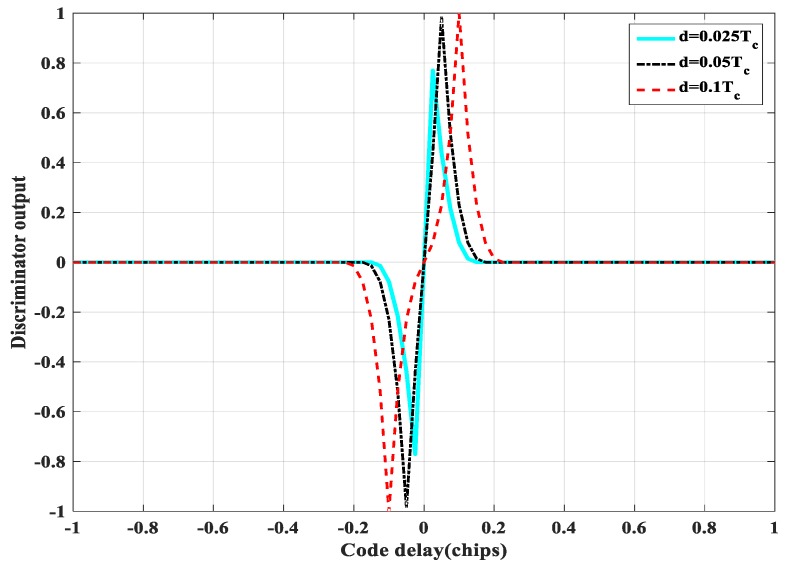
The discriminator curve at different delay spacings of BOC(1, 1).

Figure 17 shows the discriminator curve for the proposed method at different delay spacings. Compared with Figure 4, the proposed method successfully removes the mis-lock point.

**Figure 18:**
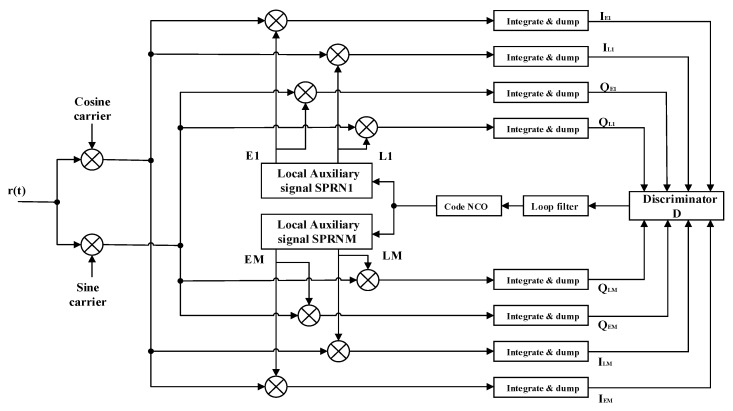
The proposed code tracking loop.

Figure 18 shows the proposed code tracking loop structure. The received intermediate frequency signal is first subjected to carrier cancellation operation. At the same time, the code loop generates two local auxiliary codes and performs Early and Late delays. The received signal is correlated with the local code. Then the local code phase is adjusted by the phase detector and the NCO (Numerically Controlled Oscillator) to complete the unambiguous tracking of the BOC signal.

### 4.2. The Code Tracking Error Analysis

The code tracking error formula is given in [18].

(38) $$\sigma_{cte}^{2}=\frac{2B_{L}\left(1-0.5B_{L}T_{coh}\right)T_{coh}\sigma_{v}^{2}}{K_{v}^{2}}$$

where $B_{L}$ is the code loop filter bandwidth, $\sigma_{v}$ is the discriminator output standard deviation, and $K_{v}$ is the discriminator gain [19].

(39) $$K_{V}={\frac{dD_{SRSA}}{d\Delta \tau }|}_{\Delta \tau =0}=16\pi P\left[\begin{array}{l} \int_{-\infty }^{\infty }fH\left(f\right)G_{B/P1}\left(f\right)\sin\left(\pi fd\right)df\int_{-\infty }^{\infty }fH\left(f\right)G_{B/PM}\left(f\right)\cos\left(\pi fd\right)df\\ -\int_{-\infty }^{\infty }fH\left(f\right)G_{B/PM}\left(f\right)\sin\left(\pi fd\right)df\int_{-\infty }^{\infty }fH\left(f\right)G_{B/P1}\left(f\right)\cos\left(\pi fd\right)df \end{array}\right]$$

In Equation (39), H(f) is the power spectral density of the front-end filter, and $G_{B/P}$ represents the cross-power spectral density between the BOC signal and the auxiliary signal. For convenience, let

(40) $$\{\begin{array}{c} V_{IE}=\left|I_{E1}I_{EM}\right|+I_{E1}I_{EM}\\ V_{IL}=\left|I_{L1}I_{LM}\right|+I_{L1}I_{LM}\\ V_{QE}=\left|Q_{E1}Q_{EM}\right|+Q_{E1}Q_{EM}\\ V_{QL}=\left|Q_{L1}Q_{LM}\right|+Q_{L1}Q_{LM} \end{array}$$

then

(41) $$D_{SRSA}=V_{IE}-V_{IL}+V_{QE}-V_{QL}$$

when the tracking process is in a steady state, considering that Δf≈0, Δφ≈0 and Δτ≈0, we have

(42) $$\begin{array}{l} E\left(D_{SRSA}\right)=0\\ E\left[\left(V_{IE}-V_{IL}\right)\cdot \left(V_{QE}-V_{QL}\right)\right]=0\\ \sigma_{V}^{2}=E\left(D_{SRSA}^{2}\right)=E\left[{\left(V_{IE}-V_{IL}\right)}^{2}\right]+E\left[{\left(V_{QE}-V_{QL}\right)}^{2}\right] \end{array}$$

As long as $E\left[{\left(V_{IE}-V_{IL}\right)}^{2}\right]$ and $E\left[{\left(V_{QE}-V_{QL}\right)}^{2}\right]$ are solved, $\sigma_{V}^{2}$ can be obtained. It can be calculated using the eigenfunction of the joint Gaussian distribution.

(43) $$\begin{array}{cc} E\left[{\left(V_{IE}-V_{IL}\right)}^{2}\right] & =2E\left[{\left(I_{E1}I_{EM}\right)}^{2}\right]+2E\left[{\left(I_{L1}I_{LM}\right)}^{2}\right]\\ & -2E\left[\left|I_{E1}I_{EM}\right|\cdot \left(I_{E1}I_{EM}\right)\right]-\;2E\left[\left|I_{L1}I_{LM}\right|\cdot \left(I_{L1}I_{LM}\right)\right]\\ & -2E\left[\left|I_{E1}I_{EM}\right|\cdot \left|I_{L1}I_{LM}\right|\right]-\;2E\left[\left(I_{E1}I_{EM}\right)\cdot \left(I_{L1}I_{LM}\right)\right]\;\;\;\;\\ & +2E\left[\left(I_{E1}I_{EM}\right)\cdot \left|I_{L1}I_{LM}\right|\right]+2E\left[\left|I_{E1}I_{EM}\right|\cdot \left(I_{L1}I_{LM}\right)\right]\;\;\;\\ & \approx 2E\left[{\left(I_{E1}I_{EM}\right)}^{2}\right]+2E\left[\left(I_{E1}I_{EM}\right)\right]{\;}^{2\;\;\;}\\ & =2\left[\left(1+\rho_{0}^{2}\right)\sigma_{0}^{4}+\cos^{2}\left(\Delta \varphi \right)\left(\mu^{2}\left(1\right)+\mu^{2}\left(3\right)+2\mu \left(1\right)\mu \left(3\right)\rho_{0}\right)\sigma_{0}^{2}\right]\;\;\;\;\;\;\;\;\;\;\;\;\;\;\; \end{array}$$

(44) $$\begin{array}{cc} E\left[{\left(V_{QE}-V_{QL}\right)}^{2}\right] & \approx 2E\left[{\left(Q_{E1}Q_{EM}\right)}^{2}\right]+2E\left[\left(Q_{E1}Q_{EM}\right)\right]{\;}^{2\;\;\;}\\ & =2\left[\left(1+\rho_{0}^{2}\right)\sigma_{0}^{4}+\sin^{2}\left(\Delta \varphi \right)\left(\mu^{2}\left(1\right)+\mu^{2}\left(3\right)+2\mu \left(1\right)\mu \left(3\right)\rho_{0}\right)\sigma_{0}^{2}\right] \end{array}$$

In Equations (43) and (44), in the case where ${\hat{R}}_{B}\left(0\right)$ is approximately equal to ${\hat{R}}_{P1/PM}\left(0\right)$, $\rho_{0}={\hat{R}}_{B/PM}^{2}\left(0\right)/{\hat{R}}_{B/P1}^{2}\left(0\right)$, $\sigma^{2}=\frac{N_{0}}{T_{coh}}{\hat{R}}_{B}\left(0\right)$.

Substituting Equations (39), (43), and (44) into Equation (38), the code tracking error variance of the proposed algorithm can be derived.

### 4.3. Performance Simulation and Analysis

Based on the Matlab platform, the intermediate frequency of the input signal is 4.092 MHz, the sampling rate is set to 40.92 MHz, the code phase offset is in the 609th sampling point, the Doppler search range is [−10 kHz, 10 kHz], and the step is 500 Hz. The four methods of SRSA, SCPC, ASPeCT and BPSK-LIKE are compared to the performance.

#### 4.3.1. Phase Discrimination Curve Analysis

**Figure 19:**
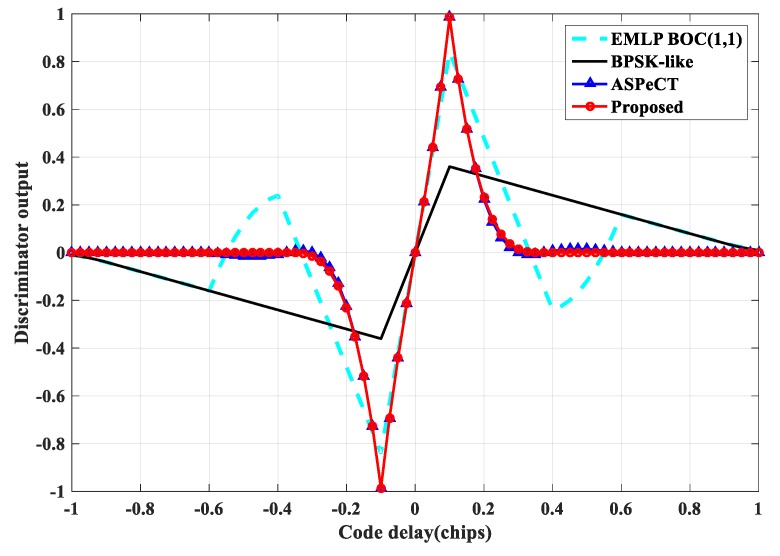
Discriminator output curve of BOC(1, 1).

**Figure 20:**
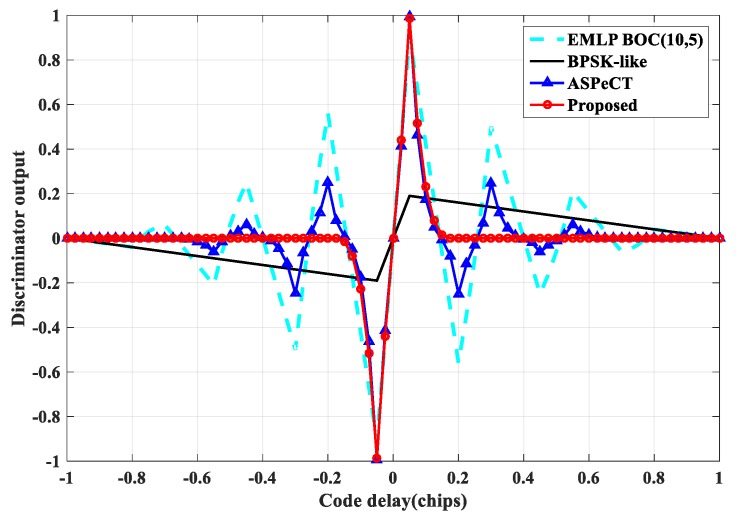
Discriminator output curve of BOC(10,5).

The classical EMLP (Early-Minus-Late-Power) phase detector is often used to generate the phase discrimination curve to evaluate the tracking performance of each method [11]. Assuming that the front-end bandwidth is infinite, compare the traditional Delay Lock Loop (DLL), BPSK-like, ASPeCT, and SRSA methods. The results show that for the second-order BOC signal (Figure 19), the phase discrimination curve of the traditional DLL has two mis-locking points, and the other three algorithms can remove the mis-locking point. The stable region is [−0.1 Tc, +0.1 Tc]. However, the slope gain of the phase discrimination curve of the DLL, ASPeCT, and SRSA methods relative to the linear region of the phase discrimination curve of BPSK-like is 5.2 dB. For the 4th-order BOC signal (Figure 20), the phase discrimination curve of the traditional DLL has six mis-locking points. The stability of the other three algorithms is [−0.05 Tc, +0.05 Tc], but the ASPeCT method has four mis-locking points, which cannot remove the tracking ambiguity. The proposed SRSA method can completely remove the mis-locking point, and the slope gain is 7.2 dB compare with BPSK-like, and the slope of the phase discrimination curve can be kept large. The phase discrimination error output is proportional to the slope of the phase discrimination curve at the center zero-crossing point, and the performance such as anti-noise and tracking jitter accuracy is closely related to this.

#### 4.3.2. Anti-Multipath Performance Analysis

**Figure 21:**
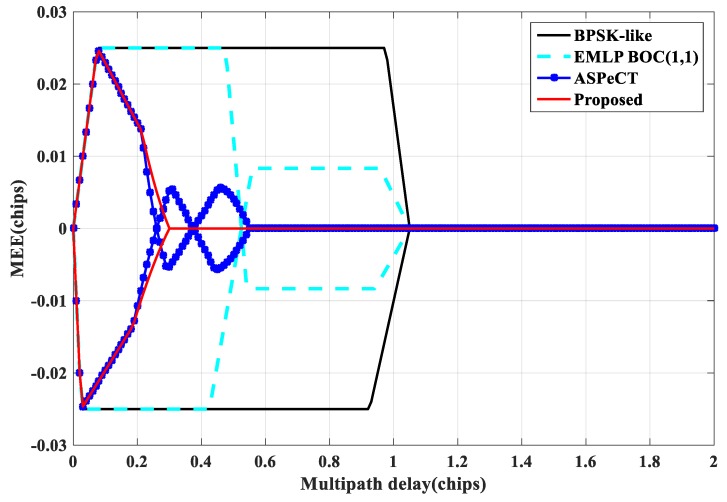
Multipath error envelope of BOC(1, 1).

**Figure 22:**
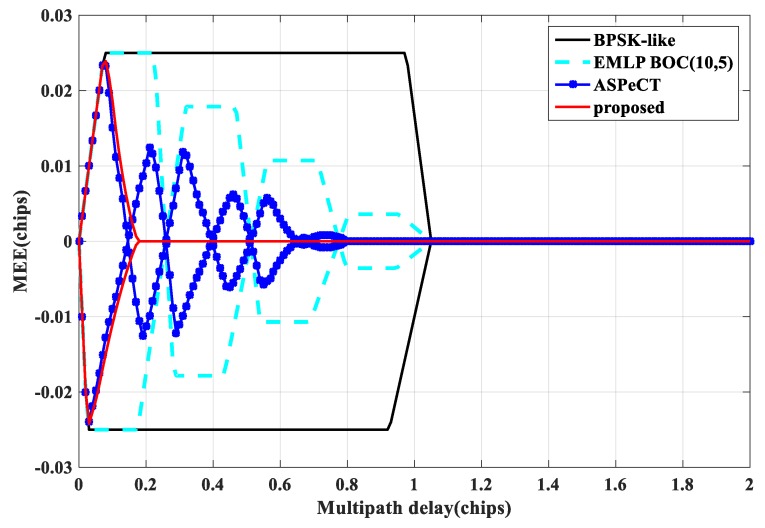
Multipath error envelope of BOC(10,5).

Multipath Error Envelope (MEE) is a typical indicator for evaluating the multipath performance of tracking loops, reflecting the sensitivity of a code tracking loop to different parameters of multipath signals [20,21]. Figure 21 and Figure 22 show the multipath envelope comparison of BOC(1, 1) and BOC(10, 5) obtained by the four tracking algorithm when the correlator interval is set to 0.1 Tc and 0.05 Tc. The envelope extreme value (the absolute maximum value of the MEE), the length of the envelope interval length (the sum of the abscissa intervals when the MEE takes a non-zero error), and the envelope area (the area enclosed by the MEE) are three measures of anti-multipath performance [22]. The smaller the three indicators, the better the anti-multipath performance. It can be seen from the Figure 21 and Figure 22 that the SRSA and ASPeCT methods have better anti-multipath performance under short multipath delay conditions. Still, ASPeCT does not completely remove the sub-peaks and causes errors in a certain multipath delay range. Therefore, the method proposed in this paper has excellent anti-multipath performance.

#### 4.3.3. Anti-Noise Performance Analysis

**Figure 23:**
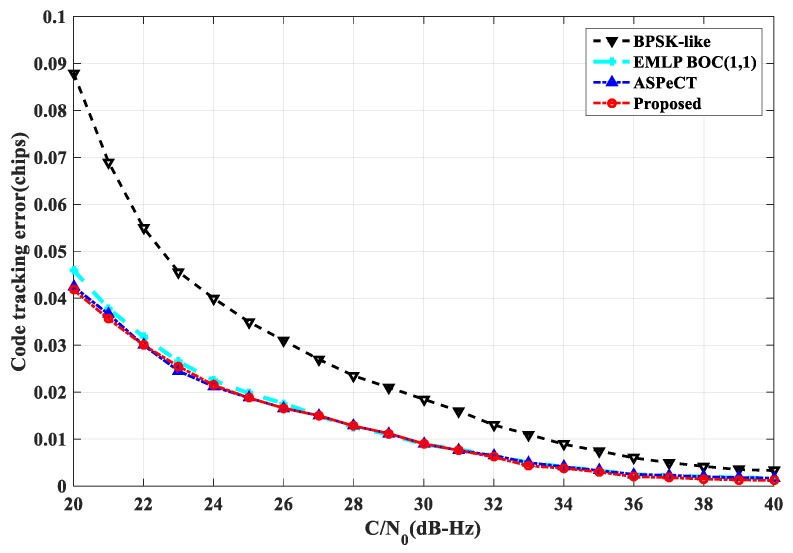
Code tracking error standard deviation of BOC(1,1).

**Figure 24:**
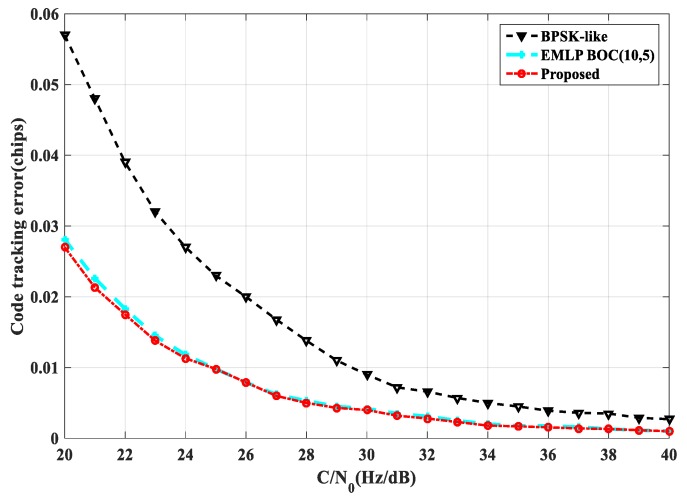
Code tracking error standard deviation of BOC(10, 5).

Thermal noise is another important cause of tracking error, and loop code tracking error is an important indicator to measure the anti-noise performance of tracking methods [23]. As shown in Figure 23, under different carrier-to-noise ratios, the single-sideband loop bandwidth is set to B=2 Hz, the coherent integration time is $T_{coh}=1\;\mathrm{ms}$, and the tracking interval is 0.1 Tc. The standard deviation of the code tracking error is obtained by tracking the BOC(1,1) using several tracking methods. The code tracking error of ASPeCT and SRSA is improved compared with the traditional EMLP phase discriminator at a low carrier-to-noise ratio, and the BPSK-LIKE method has poor code tracking error. And compared with BPSK-LIKE, the standard deviation of code tracking error is reduced by 0.045 Tc. As shown in Figure 24, under different carrier-to-noise ratios, the single-sideband loop bandwidth is set to B=2 Hz, the coherent integration time is $T_{\mathrm{coh}}=1\;\mathrm{ms}$, and the tracking interval is 0.05 Tc. The code tracking error standard deviation is obtained by tracking the BOC(10,5) by several tracking methods. Because ASPeCT is only applicable to 2nd-order BOC signals, only BPSK-LIKE, EMLP, and SRSA are analyzed here. The performance of the SRSA method is close to that obtained by EMLP, compared with BPSK-LIKE, the standard deviation of code tracking error is reduced by 0.03 Tc. The BPSK-LIKE method is still poor.

## 5. Conclusions

Aiming at the ambiguity of BOC signal acquisition/tracking, many methods have been proposed [1,2,3,4,5,6,7,8,9,10,11,12,13,14,15,16,17,18,19,20,21,22,23,24,25,26,27,28,29,30,31]. Sub-function reconstruction synchronization algorithm is proposed in this paper. By designing two auxiliary codes and performing cross-correlation operation with BOC signal, and then obtaining a single peak correlation function by Formula (17), we achieved the purpose of eliminating ambiguity. In the acquisition stage, the acquisition probability, computational complexity, and peak-to-average ratio were evaluated. Compared with ASPeCT, BPSK-LIKE, and SCPC, the SRSA method has excellent performance in the acquisition process. The SRSA method preserves the narrow correlation of the BOC signal correlation function and also removes the interference of the sub-peak. In the tracking stage, the tracking loop uses incoherent discriminator function (36) to eliminate ambiguity. The SRSA method removes the mis-locking point. In terms of anti-multipath performance, the SRSA method has the smallest envelope interval length and envelope area compared to ASPeCT and BPSK-LIKE. In terms of anti-noise performance, the SRSA method has almost the same code tracking error as the ASPeCT method and is superior to the BPSK-LIKE method. In summary, the proposed method can completely remove the threat of ambiguity while achieving significant performance advantages in terms of acquisition and tracking.

The main contributions of this paper are as follows:

(1) A sub-function reconstruction synchronization algorithm is proposed. Design auxiliary code and perform cross-correlation operation with BOC signal to obtain cross-correlation functions, as shown in Figure 7 or Figure 8. The first sub-function is at the same position as the main peak of the last sub-function and the sub-peak corresponds to 0 point. Based on this characteristic, combining Equation (17), we can obtain a new function with a single peak.
(2) The proposed method can be applied to BOC signals of arbitrary modulation order.
(3) Reduced computational complexity and resource consumption.

## Figures and Tables

**Table 1:** Comparison calculation between four algorithms.

Number	Algorithm Name	Multiplication/Times	Addition/Times
1	BPSK-Like	10Nlog_2_N + 10N	17.5Nlog_2_N + 7N
2	ASPeCT	10Nlog_2_N + 9N	17.5Nlog_2_N + 7N
3	SCPC	10Nlog_2_N + 10N	17.5Nlog_2_N + 7N
4	SRSA	10Nlog_2_N + 9N	17.5Nlog_2_N + 7N

## References

[1] Shen F., Xu G., Li Q. (2015). Non-Coherent Unambiguous Tracking Method for Cosine-BOC Signals Based on an S-Curve Shaping Technique. IEEE Signal Process. Lett..

[2] Hodgart M.S., Blunt P.D., Unwin M. The optimal dual estimate solution for robust tracking of binary offset carrier (BOC) modulation. Proceedings of the ION GNSS 2007.

[3] Hodgart M.S., Simons E. Improvements and additions to the double estimation technique. Proceedings of the Satellite Navigation Technologies and European Workshop on GNSS Signals and Signal Processing (NAVITEC).

[4] Fine P., Wilson W. Tracking algorithm for GPS offset carrier signals. Proceedings of the ION NTM 1999.

[5] Burian A., Lohan E.S., Renfors M. BPSK-like Methods for Hybrid-Search Acquisition of Galileo Signals. Proceedings of the IEEE International Conference on Communications.

[6] Julien O., Macabiau C., Cannon M.E., Lachapelle G. (2011). ASPeCT: Unambiguous sine-BOC(n,n) acquisition/tracking technique for navigation applications. IEEE Trans. Aerosp. Electron. Syst..

[7] Liu Z., Xu B., Tang X. Multipath mitigating technique for BOC(kn, n) signal in GNSS. Proceedings of the International Conference on Wireless Communications and Signal Processing.

[8] Yao Z., Lu M., Feng Z. (2010). Unambiguous sine-phased binary offset carrier modulated signal acquisition technique. IEEE Trans. Wirel. Commun..

[9] Yao Z., Cui X., Lu M., Feng Z. (2010). Pseudo-correlation-function-based unambiguous tracking technique for sine-BOC signals. IEEE Trans. Aerosp. Electron. Syst..

[10] Feng S., Xu G.H., Fang H.Y. (2015). Synthesized correlation function based unambiguous acquisition technique for sin-BOC/MBOC modulated signals. J. Syst. Eng. Electron..

[11] Yan T., Wei J., Tang Z., Qu B., Zhou Z. (2015). Unambiguous Acquisition/Tracking Technique for High-Order Sine-Phased Binary Offset Carrier Modulated Signal. Wirel. Pers. Commun..

[12] Ji Y.-F., Liu Y., Zhen W.-M., Sun X.-Y., Yu B.-G. (2017). An unambiguous acquisition algorithm based on unit correlation for BOC(n,n) signal. IEICE Trans. Commun..

[13] Wang C., Yang C., Ku S., Liu C. A Low-Complexity Peak-to-Average Power Ratio Estimation Method for OFDM Signals. Proceedings of the IEEE Vehicular Technology Conference.

[14] Wu J., Jiang L., Chen Y., Senhadji L., Shu H. A generalized modified split-radix FFT algorithm for N = q × 2^m^ and its applications. Proceedings of the IEEE 2014 7th International Congress on Image and Signal Processing.

[15] Lei D., Lu W., Yu D. (2015). Resource-efficient acquisition architecture for BOC-modulated signals. IEICE Electron. Express.

[16] Zhou Y., Hu X., Ke T., Tang Z. (2012). Ambiguity Mitigating Technique for Cosine-Phased Binary Offset Carrier Signal. IEEE Trans. Wirel. Commun..

[17] Yan T., Wei J., Tang Z., Qu B., Zhou Z. (2015). Unambiguous combined correlation functions for sine-BOC signal tracking. GPS Solut..

[18] Kao T.L., Juang J.C. (2012). Weighted discriminators for GNSS BOC signal tracking. GPS Solut..

[19] Li T., Tang Z., Wei J., Zhou Z., Wang B. (2019). Unambiguous Tracking Technique Based on Combined Correlation Functions for Sine BOC Signals. J. Navig..

[20] Mao H., Wu D. (2016). Evaluation of Anti-multipath Performance to Satellite Navigation Signal Tracking on L1/E1/B1 Frequency Band. J. Syst. Simul..

[21] Deng Z., Hu E., Yin L., Liu W. (2018). An Unambiguous Tracking Technique for Sine-BOC(kn,n) Modulated GNSS Signals. Wirel. Pers. Commun..

[22] Liu H.C., Cheng X., Ni S.J. (2011). Evaluation of Multipath Mitigation Performances Based on Error Envelope. J. Natl. Univ. Def. Technol..

[23] Wu J., Dempster A.G. (2012). Code Tracking Variance Analysis for GNSS Receivers with “Strobe Correlators”. IEEE Trans. Aerosp. Electron. Syst..

[24] Borio D. (2017). Coherent side-band BOC processing. IET Radar Sonar Navig..

[25] Feng T., Kai Z., Liang C. (2016). Unambiguous Tracking of BOC Signals Using Coherent Combination of Dual Sidebands. Comput. Educ..

[26] Li T., Wei J.L., Tang Z.P., Zhou Z.H., Wang B.Y. An Optimizing Combined Unambiguous Correlation Functions for BOC Signals Tracking. Proceedings of the 2017 International Technical Meeting of the Institute of Navigation.

[27] Zhang H., Ba X., Chen J., Zhou H. (2017). Unambiguous acquisition technique for BOC(m,n) modulated signals. Acta Aeronaut. Astronaut. Sin..

[28] Shim D.-S., Jeon J.-S. (2018). An Unambiguous Delay-And-Multiply Acquisition Scheme for GPS L1C Signals. Sensors.

[29] Xu C., Liu Z., Tang X., Wang F. (2016). A Design Method of Code Correlation Reference Waveform in GNSS Based on Least-Squares Fitting. Sensors.

[30] Zhen L., Jie H., Wang J., Zhao Y., Chen S.W. (2017). Generalized unambiguous tracking method based on pseudo correlation function for multi-level coded symbol modulated signals. Acta Phys. Sin..

[31] Zhen L., Zhang J.Y., Lu M.Q., Jie H., Zhao Y.J. (2017). Universal evaluation criteria for code delay estimation error of satellite navigation signals. Acta Phys. Sin..

